# Proliferative Diabetic Retinopathy Diagnosis Using Varying-Scales Filter Banks and Double-Layered Thresholding

**DOI:** 10.3390/diagnostics13132231

**Published:** 2023-06-30

**Authors:** Noor ul Huda, Anum Abdul Salam, Norah Saleh Alghamdi, Jahan Zeb, Muhammad Usman Akram

**Affiliations:** 1 Center for Advanced Studies in Telecommunications (CAST), COMSATS Institute of Information Technology, Islamabad 45550, Pakistan; 2 Computer and Software Engineering Department, College of Electrical and Mechanical Engineering, National University of Sciences and Technology, Islamabad 24090, Pakistan; anum.abdulsalam@ceme.nust.edu.pk (A.A.S.);; 3 Department of Computer Sciences, College of Computer and Information Sciences, Princess Nourah bint Abdulrahman University, P.O. Box 84428, Riyadh 11671, Saudi Arabia

**Keywords:** image processing, biomedical imaging, diabetic retinopathy, proliferative diabetic retinopathy, autonomous disease detection, fundus image analysis

## Abstract

Diabetic retinopathy is one of the abnormalities of the retina in which a diabetic patient suffers from severe vision loss due to an affected retina. Proliferative diabetic retinopathy (PDR) is the final and most critical stage of diabetic retinopathy. Abnormal and fragile blood vessels start to grow on the surface of the retina at this stage. It causes retinal detachment, which may lead to complete blindness in severe cases. In this paper, a novel method is proposed for the detection and grading of neovascularization. The proposed system first performs pre-processing on input retinal images to enhance the vascular pattern, followed by blood vessel segmentation and optic disc localization. Then various features are tested on the candidate regions with different thresholds. In this way, positive and negative advanced diabetic retinopathy cases are separated. Optic disc coordinates are applied for the grading of neovascularization as NVD or NVE. The proposed algorithm improves the quality of automated diagnostic systems by eliminating normal blood vessels and exudates that might cause hindrances in accurate disease detection, thus resulting in more accurate detection of abnormal blood vessels. The evaluation of the proposed system has been carried out using performance parameters such as sensitivity, specificity, accuracy, and positive predictive value (PPV) on a publicly available standard retinal image database and one of the locally available databases. The proposed algorithm gives an accuracy of 98.5% and PPV of 99.8% on MESSIDOR and an accuracy of 96.5% and PPV of 100% on the local database.

## 1. Introduction

Diabetic retinopathy (DR) is a vascular complication of eye. It is the most severe abnormality among all diabetic eye diseases [1]. In DR, at first a lesion starts to appear on the retina, and then it results in the bleeding of blood vessels and capillaries on the surface of the retina. Due to the leakage of blood vessels, the oxygen supply to the retina decreases and as a consequence the brain stimulates the formation of new blood vessels to fulfill the requirement of oxygen. These new vessels may bleed and cause the detachment of the retina and ultimately loss of vision [2,3]. Out of all DR patients, few suffer from proliferative diabetic retinopathy (PDR), but if it is not diagnosed in a timely manner, the disease may cause severe destruction. Neovascularization appears as a tortuous collection of blood vessels and is quite destructive because these vessels grow abnormally out of the retina into the clear vitreous gel [4,5]. Therefore, vessels grow beyond the supporting structure of the retina and they are very disposed to bleeding, particularly when they arise near the optic disc. Even a small rise in blood pressure can cause hemorrhages in this case. If bleeding appears in the vitreous humor it can affect the visual system. If this bleeding becomes extensive, it results in a painless and rapid blackening of the vision. Neovascularization [6] is divided into two types [7]:

- Neovascularization on disc (NVD): If the new vessel formation occurs within one disc diameter of the optical disc then this is categorized as NVD or neovascularization on disc
- Neovascularization elsewhere (NVE): If new vessel formation occurs elsewhere on the surface of the retina, then this is called neovascularization elsewhere (NVE).

**Figure 1:**
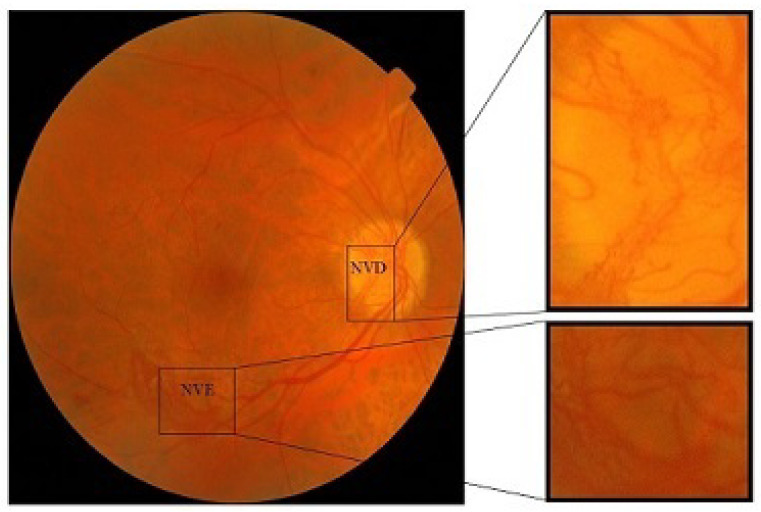
Digital fundus image with NVD and NVE.

Figure 1 highlights the classification of neovascularization. The article consists of five sections. Section 2 of the paper describes the existing work and the main contributions presented by the proposed method. The details of the proposed system are given in Section 3. Section 4 shows the results and evaluation while Section 5 is composed of the conclusion and future work.

## 2. Related Work

Several techniques have been proposed so far for the automated screening and detection of non-proliferative diabetic retinopathy and its stages [8,9,10], However, for detection of PDR a very few work has been carried out. We have organized the related literature based on two subcategories, i.e., PDR detection using machine learning techniques and PDR detection using deep learning techniques.

### 2.1. Machine-Learning-Based Algorithms

Gotman et al. [11] proposed a method for the detection of new vessels on the disc using a support vector machine (SVM) classifier. They used a watershed transform combined with 2D Gaussian for the segmentation of blood vessels. Fifteen features were used for the classification of the SVM classifier and they achieved an area under receiver operating characteristics curve (ROC) of 0.909. They came up with a feature set to detect neovascularization but they limited their scope to NVD only. An amplitude modulation–frequency modulation (AM-FM)-based method was proposed by Agurto et al. [12]. They followed a top-down approach for the detection of NVD. They came up with a sound set of features and used K-means clustering on these feature sets. Jelinek et al. [13] proposed a new technique for the detection of proliferative diabetic retinopathy from angiograms. They used 27 labeled images and achieved an accuracy of 0.90 with the selection of six features. Derivatives of the Gaussian wavelet were used in their work for the segmentation of blood vessels. Mudigonda et al. [14] proposed a method for the detection of neovascularization in retinal fundus images using fractal analysis. The proposed technique used colored fundus images as the input, followed by ROI extraction, i.e., extraction of the region around the optic disc. The green channel was extracted from the ROI region to obtain the maximum information from the vessels. The vessels were extracted using a Gabor filter, and the resulting magnitude image was converted to a binary image. The resultant image was analyzed using a fractal analysis box-counting method that identified vessel bundles near the optic disc region, i.e., neovascularization in the optic disc region (NVD). Among ten images, five with neovascularization had a fractal mean value of 1.66 and five images with no neovascularization in the optic disc region resulted in a fractal mean value of 1.58. Saranya et al. [15] used the fuzzy C-means (FCM) technique for blood vessel segmentation. They used a set of features that included the gradient, gradient variation, gray-level coefficient of variation, moment invariant-based features, and tortuosity for a k-nearest neighbor (KNN) classifier. They achieved an accuracy of 96.5% on the DRIVE and MESSIDOR datasets. A research methodology for the automated detection of neovascularization for PDR proposed by Sohini Roy Chowdhury et al. [16] describes a technique to detect neovascularization from fundus images and classify it as neovascularization in the optic disc region (NVD) or neovascularization elsewhere (NVE). The green plane is extracted from the input image and normalized in the [0,1] intensity range. The region of interest (ROI) is extracted for both types of neovascularization, leading to vessel detection from both ROIs. Textural, structural, and intensity-based features are used to classify NVD and NVE. The proposed method was trained and tested on 40 images (30 normal, ten with PDR) from the STARE database and 17 images from a local dataset. Accuracies of 87.6% and 92.1% were obtained for NVD and NVE, respectively.

Lee et al. [17] proposed a new vessel-detection method that includes statistical texture analysis (STA), high-order spectrum analysis (HOS), and fractional analysis. They used a total of 137 images in their work and achieved an area under the curve of 99.3%. A method based on the following-the-line approach for the segmentation of vessels was proposed by R.A. Welikala et al. [18]. They used two-line approaches and two different sets of features for the retraction of true abnormal blood vessels. They used 60 images from the MESSIDOR dataset for evaluation purposes and classified these images on the basis of an SVM classifier. They achieved an area under the curve (AUC) value of 0.96. Shuang Yu et al. [19] proposed a novel technique for the automation of neovascularization in the optic disc region (NVD). A fundus image is pre-processed followed by the application of a Gabor filter to extract blood vessels. Twenty-one texture-based and vessel-based features are extracted to classify an image as normal or NVD using support vector machines (SVM). Sixty-six retinal images (15 NVD, 50 normal) were extracted from the globally available MESSIDOR, HRF, and DIARETDB0 datasets to test and train the proposed technique. A sensitivity of 15/16 and specificity of 47/50 were achieved using the proposed methodology.

In 2016 research was conducted by Diego F. G. Coelho [20] that aimed to detect NVD from fundus images. In the proposed methodology, fundus images are analyzed by calculating the gradient magnitude of the Fourier power spectrum followed by extraction of the angular spread. Entropy and spatial variance are used to categorize an image as a normal image or image with NVD using a linear statistical classifier. An accuracy of 100% was achieved when the proposed technique was tested on ten images (five normal, five NVD) extracted from the MESSIDOR database. Akram et al. [7] presented a method for the detection of PDR. Their proposed method extracted a number of features based on vascular patterns for the proper representation of normal and abnormal vessels. A modified m-mediods-based classifier was used for the proper discrimination of abnormal vessels from normal ones. Another machine-learning-based technique for automated NVD detection was proposed by Shuang Yu et al. [21]. The proposed algorithm takes a fundus image and extracts the ROI, i.e., the disc region. Vessels are extracted using multilevel Gabor filters. A feature vector with 42 features (morphological and texture based) is extracted from both normal and NVD images, followed by a reduction in size to eighteen. A reduced feature vector was used to train and test 424 (134 NVD, 290 non-NVD) retinal fundus images. An accuracy of 95.23% was observed.

Christodoulidis et al. [22] proposed a novel technique for the detection of NVD from fundus images. The proposed research states that NVD detection from retinal fundus images can be improved by adding a second-order statistical feature to the existing feature set containing structural, vessel-based, and intensity-based features. The image is pre-processed, followed by vessel detection. The vessel junctions are extracted by applying the Tensor voting technique, which highlights the local maxima, indicating the junctions of vessels. The suggested feature addition to the feature vector improved the sensitivity to 0.84. Mona Leeza et al. [23] proposed an algorithm in 2019 to detect the severity level of diabetic retinopathy using the bag-of-features approach. The algorithm is composed of five phases starting from local feature extraction from retinal images using SURF. K-means clustering is used to cluster the extracted features for dictionary generation. The algorithm proceeds by max pooling to accumulate features followed by the construction of histograms of oriented gradient (HoG). SVM and artificial neural networks are used to classify the retinal image as normal, mild NPDR, moderate NPDR, severe NPDR, and PDR. The algorithm resulted in 95.92% and 98.90% sensitivity and specificity, respectively. Research conducted by Lei Zhang et al. [24] described another algorithm to screen for PDR using a modified matched-filter approach. In the proposed technique, the result from Gaussian is proceeded by subtraction of the mean to eliminate the false positives that occur due to step edge noise. The accuracy of the algorithm was evaluated on the ZUEYE database, which resulted in an accuracy of 95%.

### 2.2. Deep-Learning-Based Algorithms

A technique was proposed in 2019 [25] to detect and categorize diabetic retinopathy using a deep convolution neural network (CNN) of five layers. The algorithm starts with pre-processing of the images, followed by an ensemble CNN model. The ensemble CNN model is composed of five deep CNN models, i.e., Resnet50, Inceptionv3, Xception, Dense121, and Dense169. The CNN model classifies the input image as normal, mild, moderate, severe, or PDR. The algorithm was tested on the Kaggle dataset, composed of 35126 colored retinal images. Sixty-four percent of images in the dataset were used for training, 20% images were used for testing, and 16% were used for validation. Specificities of 0.40, 0.99, 0.95, 0.98, and 0.99 were observed for each category, respectively. In 2022 [26], a neural-network-based framework was proposed using optical coherence tomography (OCT) images. The proposed algorithm classified an OCT image as normal or diseased using 3D feature extraction. Initially, segmentation was performed to extract 12 layers from the input image followed by feature extraction, i.e., thickness and angle calculation. The extracted features were then fused and passed to the neural network to make a decision, yielding an accuracy of 96.61%.

Ayesha et al. [27] proposed three deep neural frameworks for diabetic retinopathy grading using retinal fundus images. The first framework used cascaded architecture to grade a retinal image among five grades of PDR using a three-layer CNN architecture. The second framework utilized the hue saturation value (HSV), red green blue (RGB), and normalized input image to apply ensemble-based architecture, where the final results were deduced using average pooling from each CNN model. The third framework incorporated a long short-term memory (LSTM) module to enhance the network memorizing capabilities. The EyePACS dataset containing 88,702 retinal images was used to train and test the proposed framework. Among all, the ensemble-based architecture outperformed, resulting in an accuracy of 83.78%. Another framework proposed by Tang et al. [28] segmented and localized neovascularization using a deep learning architecture. The proposed algorithm starts with image pre-processing followed by dividing the input image into non-overlapping patches. To train the neural network, the ground truth containing neovascularization in each patch was passed as a training dataset, which classified each pixel of the patch as neo or non-neo. The dataset was divided into validation, training, and testing sets, yielding an accuracy of 0.9948 on a dataset with 50 images. This research was further extended [29] using transfer learning on pre-trained models that included AlexNet, GoogLeNet, ResNet18, and ResNet50 pre-trained on ImageNet. Ground truth patches were used for training these models, followed by testing the models. Another module utilized the pre-trained model for feature extraction followed by classification using SVM. In addition to using pre-trained models separately, a combination of ResNet and GoogleNet was proposed that yielded the highest accuracy of 0.9157.

Another algorithm [30] utilized ResNet to detect retinal neovascularization from retinal fundus images. The proposed framework pre-processed the input image to enhance the contrast and remove noise followed by training ResNet. The proposed model was trained on 3662 retinal images from a local dataset containing healthy images (Label 0), neovascularized images (Label 2), and diabetic retinopathy images (Label 1). Due to the residual properties of ResNet, the model resulted in an accuracy of 0.88 on 1992 retinal fundus images. A review conducted by Salamat et al. [6] summarized and comprehended 66 papers aiming to detect and classify diabetic retinopathy using various techniques. The paper presented the past 8 years of research articles starting from 2019, indicating the techniques used, the dataset, and the evaluation metrics along with the type of classification or number of classes. The majority of the techniques presented in the review classified an image into healthy or diseased without classifying it by the degree/severity of disease.

Our proposed method is an extension of [7]. Our method grades the PDR without the use of a classifier and utilizes a simple feature-extraction approach to minimize false-positive detection. The main issues with most of the algorithms mentioned are the appearance of false-positive regions for abnormal blood vessels and evaluation on a very small dataset. The proposed algorithm is novel in the sense that it addresses both NVD and NVE. It uses a very robust method for the detection of optic disc coordinates. It gives almost no false detection, unlike other cases. The algorithm extracts optic disc and candidate abnormal blood vessel regions using the vascular structure and filter bank, respectively. Double-layered thresholds on the basis of pattern analysis for the detection of PDR are applied, which grade the input retinal image as NVD or NVE.

## 3. Materials and Methods

**Figure 2:**
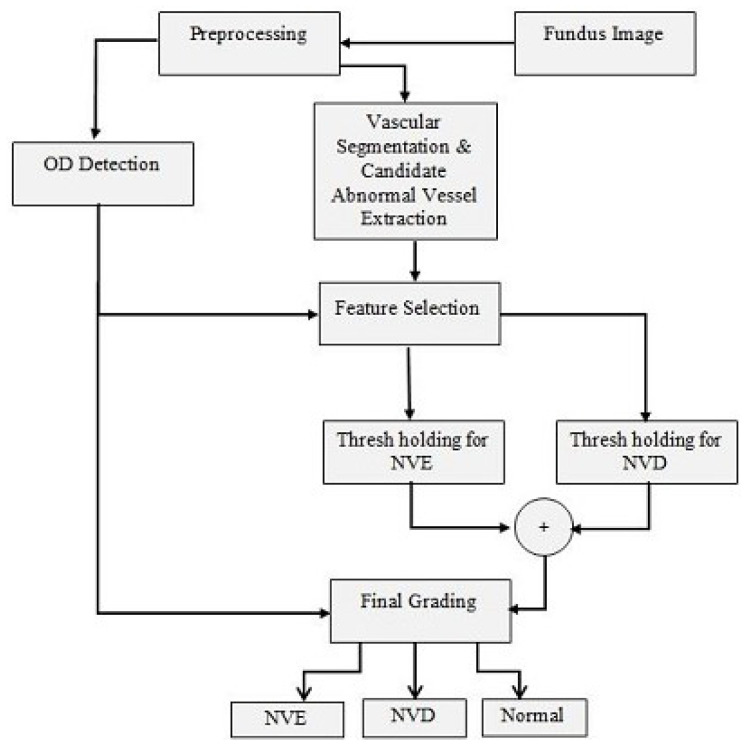
Proposed flow diagram for the detection and grading of PDR.

The proposed system follows the following steps. It first performs pre-processing on the images to enhance the vascular pattern, which is followed by blood vessel segmentation and optic disc localization. Various features are tested on the candidate regions with different thresholds for the separation of positive and negative advanced diabetic retinopathy cases. Optic disc coordinates are applied for the grading of neovascularization as NVD or NVE. The algorithms improve the quality of the automated system by eliminating normal blood vessels and exudates for the accurate detection of abnormal blood vessels. The whole process can be arranged into four basic steps, which involve pre-processing, vessel extraction, ROI processing, and post-processing. Figure 2 shows the complete flow of the automated system for grading PDR.

### 3.1. Preprocessing

**Figure 3:**
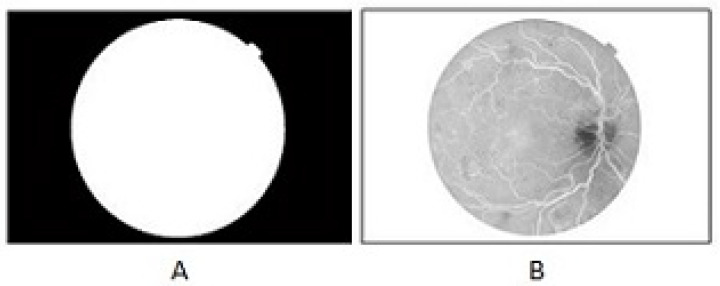
Background segmentation, (**A**) Extracted Background Mask (**B**) Background-segmented and scaled image in the inverted green channel.

An automated assessment for pathologies of the retina initially requires the pre-processing of a digital fundus image. An inverted green channel is used as it enhances the vascular patterns against the dark background. All images are scaled to the same size, not disturbing their aspect ratio. The dark background of the image is not really black as it contains some of the lighter regions and an amount of noise. It is necessary, for the proper extraction of the vascular pattern in the retina, to separate the noisy background from the image. For this purpose, background segmentation is carried out. The method first creates a binary mask for the background by using the mean and variance and then it eliminates the small noisy pixel values from the background by using different morphological operators [31]. Figure 3 shows the images after pre-processing.

### 3.2. Vessel Segmentation

After pre-processing, a 2D Gabor wavelet is applied to the image to enhance the vascular pattern, so that the abnormal blood vessels, which are thin and less visible, become visible and prominent [32]. Gabor wavelets can be set to specify a direction for vessel segmentation. They are very sensitive to small edges and have directional selectivity capability. They also act as a filter for the background noise. In this work, 2D continuous wavelet Gabor transform (CWT) is used. It is defined in (1):

(1) $$T_{\psi }\left(b,\theta ,a\right)=C_{\psi }^{-1/2}a\int \exp\left(j\mathrm{kb}\right){\hat{\psi }}^{*}\left(ar_{-\theta }k\right)\hat{g}\left(k\right)d^{2}k$$

where $j=\sqrt{-1}$, and ${\hat{\psi }}^{*}$ and $\hat{g}$ denote a Fourier transform. The 2D Gabor wavelet is defined as:

(2) $$\psi_{G}\left(x\right)=\exp\left(jk_{0}x\right)\exp(-\frac{1}{2}{\left|\mathrm{Ax}\right|}^{2})$$

(3) $${\hat{\psi }}_{G}\left(x\right)={\left(\det\left(B\right)\right)}^{1/2}\exp\left(-\frac{1}{2}\left(B{\left(k-k_{0}\right)}^{2}\right)\right)$$

where $k_{0}$$\in R^{2}$ is a vector that defines the frequency of the complex exponential, $B=A^{-1}$ and $A=\left[\begin{array}{cc} \epsilon^{-1/2} & 0\\ 0 & 1 \end{array}\right]$ with elongation ϵ≥1 is a 2×2 positive definite diagonal matrix that defines the wavelet anisotropy and its elongation in any desired direction.

The Gabor wavelet transform $M_{\psi }\left(b,a\right)$ is computed for each pixel position and its scale value is considered. In addition, θ spans from 0° to 165° at steps of 10° and its maximum is taken.

(4) $$M_{\psi }\left(b,a\right)=max\left|T_{\psi }\left(b,\theta ,a\right)\right|$$

Multilayered and adaptive thresholding techniques are applied to create a binary mask for blood vessels after the completion of blood vessel enhancement [33]. The masking process assigns 1 to all vessel pixels and 0 to all non-vessel pixels.

### 3.3. Abnormal Vessel Detection

**Figure 4:**
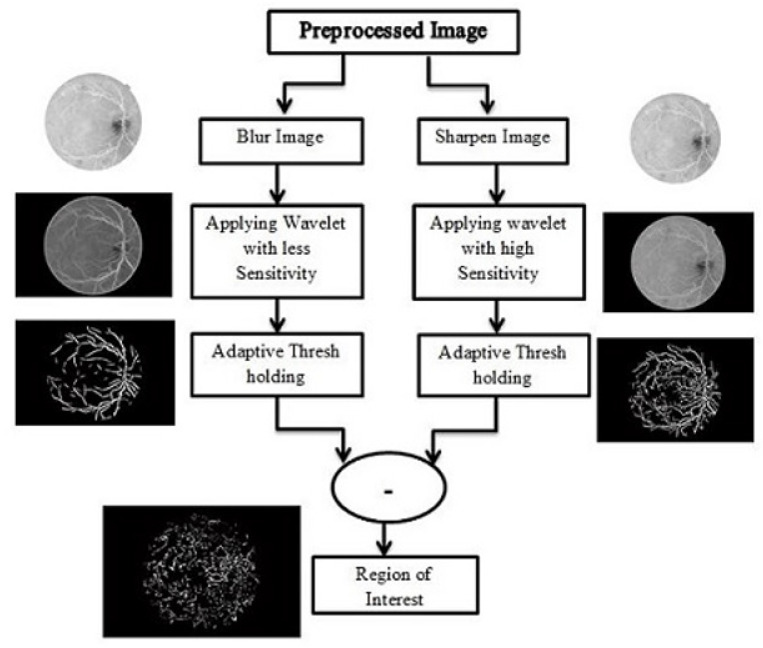
Flow for abnormal blood vessel extraction.

The abnormal blood vessel extraction process is explained in Figure 4. Two copies of the image are created after pre-processing. A Gaussian blur filter of 3 × 3 is applied to the first copy of the image. This filters out the minor details. This copy of the image is further processed using a 2D Gabor filter that is set on such frequencies and directions to enhance the normal blood vessels only. The second copy of the image is fed to a sharpening filter to enhance the small details in the image. Then a 2D Gaussian filter is applied to this copy of the image with set frequencies that also enhance the small details as well as normal blood vessels. Table 1 and Table 2 show the selected values for each parameter of Gabor wavelet for blood vessel enhancement for NVD and NVE cases, respectively.

Then multilayered thresholding is applied on both copies of the image as described in Section 2.2. Both copies are then subtracted to come up with a region that contains abnormal blood vessels and exudates only. This is our candidate region of interest.

### 3.4. Optic Disc Detection

**Figure 5:**
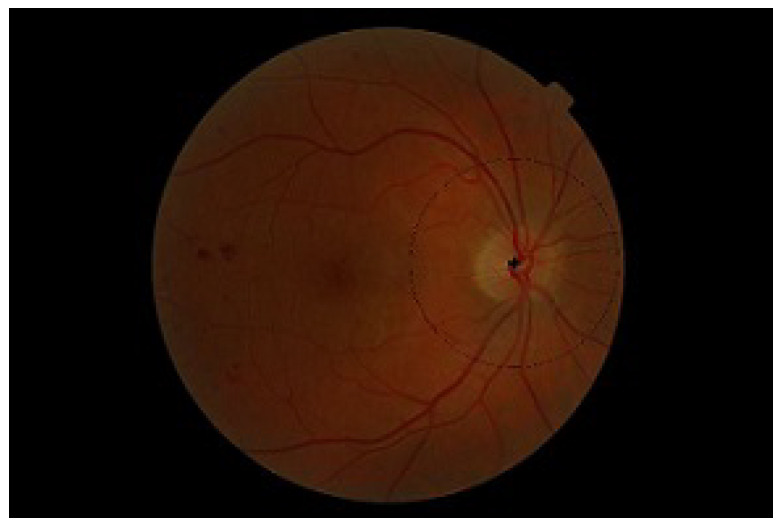
Retinal image showing the optical disc coordinates.

The optic disc (OD) is a comparatively brighter region in the fundus image with a bright yellowish color and circular shape, but it also shows some variation in brightness and color if some disease is present, which can make OD detection difficult. The optic disc (OD)-detection algorithm is basically divided into two stages. In the first stage, the optic disc is segmented, where candidate regions are calculated using a Fourier transform followed by morphological operations. If more than one candidate region appears, then blood vessel segmentation is carried out to calculate the energy of each region. The region with the maximum energy is marked as the OD [34]. Figure 5 shows the OD coordinates after applying the algorithm.

The binary thresholded image that we obtain after the abnormal blood vessel extraction contains both the lesions and the abnormal blood vessels. Neovascularization on disc (NVD) is graded as the abnormal blood vessels detected at one disc distance (1dd) from the optic disc coordinates. If D denotes the diameter of the disc then one disc diameter can be calculated using:

(5) 1dd=D+(D/2)

**Figure 6:**
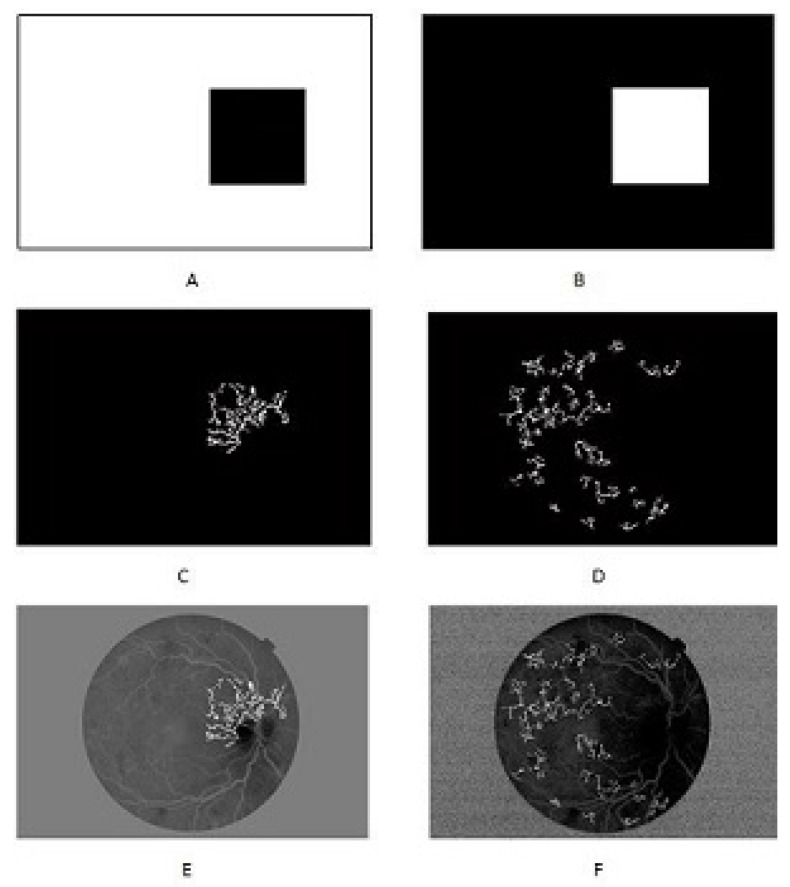
(**A**,**C**,**E**) show the filter mask, binary filtered image, and its map on the original green channel for NVD, respectively. (**B**,**D**,**F**) show the filter mask, binary filtered image, and its map on original green channel for NVE, respectively.

By applying the one-disc distance, two filter masks are created, one for the NVD case and the other for the NVE case. These masks are applied to the image for the extraction of features. Figure 6 shows the filter masks and images to be further processed. True and false objects in the binary image are first classified on the basis of the 0^*th*^ moment of the image, that is, the area of the candidate ROI. Let the characteristic function for the object in the image be L(x,y). We define:

L(x,y)=0forobjectandL(x,y)=1forbackground

so the area of the region can be defined as:

(6) A=∫∫L(x,y)

The first-order moment, i.e., the center of mass of the objects, is then calculated. Let the center of mass be denoted by $\left(\bar{x},\bar{y}\right)$. Then:

(7) $$\bar{x}=\frac{\int \;\int xL(x,y)}{\int \;\int L(x,y)}$$

(8) $$\bar{y}=\frac{\int \;\int yL(x,y)}{\int \;\int L(x,y)}$$

where *x* and *y* are the coordinates of the image. The center of mass is then chosen as the center of the window for the region of interest. Choosing an appropriate window size is quite crucial. It is chosen to include all the abnormal blood vessels.

### 3.5. Feature Selection and Thresholding

It is observed that the abnormal vessels that grow around the optic disc tend to be in large bunches. They consume more area and are more tortuous. In contrast to that, the abnormal blood vessels found elsewhere on the retina are comparatively very small and consume a relatively small area. By considering the properties of these blood vessels the feature sets used for the classification of NVD are:

- Entropy: Entropy is the measure of uncertainty in a system. Abnormal blood vessels are fragile and follow no proper pattern. Thus, the regions that contain abnormal blood vessels have a high entropy value. If $p_{k}$ is the probability of occurrence of a grey level *k* and *M* is the number of grey levels in the image, then entropy is calculated as:
  (9) $$H=-\sum_{k=1}^{M}\left(p_{k}\right)\log_{2}\left(p_{k}\right)$$
- Energy: Energy is the sum of squares of all pixel intensities within a candidate region of interest. The energy of the region containing the abnormal blood vessels lies in between those of the regions that contain normal blood vessels and the bright lesions or exudates. If g(x,y) is the pixel value in an image then the energy is calculated as:
  (10) $$E=\sum_{i,j}g{\left(i,j\right)}^{2}$$
- Homogeneity: Homogeneity returns a value that tells the closeness of the distribution of elements. The homogeneity of abnormal blood vessels lies very close to that of normal blood vessels but it is away from that of the lesions and exudates. The abnormal blood vessels originate near normal vessels, while the exudates and bright lesions can be found anywhere on the retina. The homogeneity is calculated as:
  (11) $$H=\sum_{i,j}\frac{g(i,j)}{1+|i-j|}$$

The feature sets used for the classification of NVE are:

- Energy: As a smaller window size was chosen for NVE, it shows a relatively high energy value in that small area.
- Gradient: The mean gradient magnitude in the candidate region of interest is calculated by using the Sobel gradient operator. Separate measurements of the gradient component in each orientation, called $G_{x}$ and $G_{y}$, are calculated. Then the magnitude of the gradient is given by:
  (12) $$G\left(x,y\right)=\sqrt{G_{x}^{2}+G_{y}^{2}}$$
  The mean of the gradient magnitude is used as a feature, which is:
  (13) $$M_{m}ag=\frac{1}{nm}\sum_{i,j}G\left(i,j\right)$$
  where m and n are the dimensions of the region of interest.
- Gradient Direction: The directional gradient is the standard deviation of the Sobel gradient in the candidate region of interest. As the abnormal vessels are much less defined, are less homogeneous, and have more contrast variation than normal vessels, this feature is taken in to account. The direction can be calculated as:
  (14) $$\theta \left(x,y\right)=\arctan\frac{G_{y}}{G_{x}}$$
  and the mean of its standard deviation is:
  (15) $$s=\frac{1}{m}\left(\sqrt{\frac{\left(\theta -\bar{\theta }\right)}{\left(n-1\right)}}\right)$$

The value for the feature sets is chosen inside a fixed window size for each candidate ROI. The features and characteristics of newly grown vessels lie in between those of the original vascular pattern and other false detection. Thus, thresholds are applied on both the upper and lower bounds. After thresholding, there still remains some wrongly detected candidate regions of interest, which can be called false-positive detection. A box plot analysis is used for the further analysis and processing of false-positive detection.

### 3.6. Post-Processing

The regions that are obtained after ROI processing are grouped into two classes. One contains the true positive, i.e., the region that actually has neovascularization, and the other contains the false positive, which does not have neovascularization. A set of features is applied to both classes. In this analysis, the size of the window for analysis of features is kept adaptable to the size of the ROI. These features are as below:

1. Mean Intensity $\left(f_{1}\right)$: It is the mean value of pixels within the green plane of the candidate region.
2. Maximum Intensity $\left(f_{2}\right)$: It is the maximum value of pixels within the green channel of the candidate region.
3. Mean Skewness $\left(f_{3}\right)$: It is the measure of the lack of symmetry in a candidate region. It is computed as:
  (16) $$skewness=\frac{\sum_{i=1}^{N}{\left(g_{i}-\bar{g}\right)}^{3}}{\left(n-1\right)s^{3}}$$
  where g(x,y) is the candidate pixel value, $g(\bar{x},y)$ is the mean value pixels, s is the standard deviation, and N is the number of pixels in the candidate region.
4. Entropy $\left(f_{4}\right)$: It is the value of all pixels in a candidate region and its neighboring pixels. It is the measure of unpredictability in an ROI.
5. Energy $\left(f_{5}\right)$: It is the sum of the squares of all the pixel values of the green plane inside a candidate region.
6. Mean Gradient $\left(f_{6}\right)$: It is the mean of the pixels of the edges detected using the Sobel gradient within the candidate region.
7. Gradient Direction $\left(f_{7}\right)$: It is the standard deviation of the direction of the Sobel gradient in a candidate region.
8. Mean Intensity of red plane $\left(f_{8}\right)$: It is the mean value of pixels within the red plane of the candidate region.
9. Mean Intensity of blue plane $\left(f_{9}\right)$: It is the mean value of pixels within the blue plane of the candidate region.
10. Mean Intensity lightness in LAB color space $\left(f_{9}\right)$: It is the mean value of pixels within the lightness plane in the LAB color space of the candidate region.

**Figure 7:**
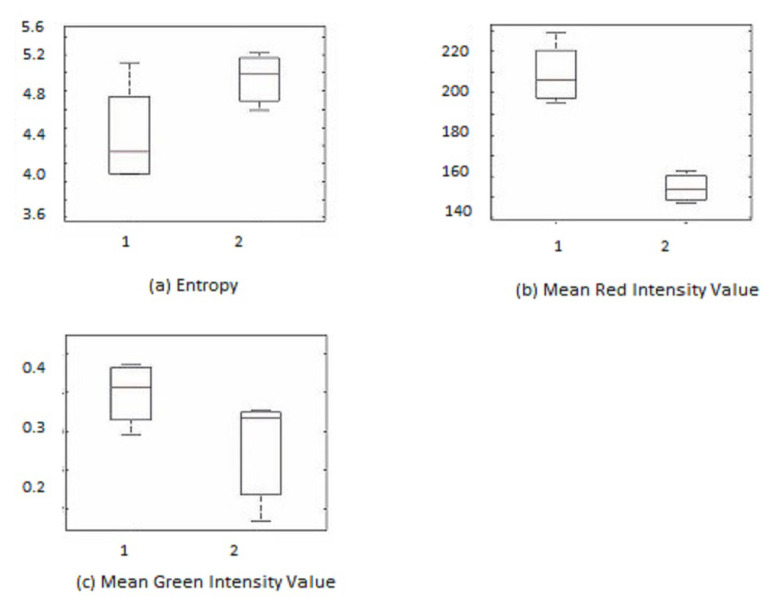
Box plots of best features for NVE. (1) True detections, (2) false detections.

**Figure 8:**
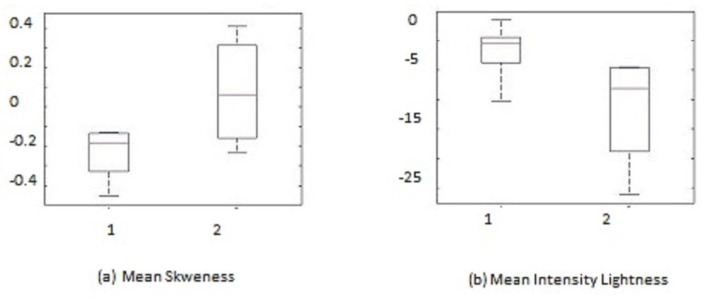
Box plots of best features for NVD. (1) True detections, (2) false detections.

A number of the features mentioned were tested on both sets of candidate ROIs, i.e., NVD and NVE. However, not all of them are useful in improving the accuracy of the cases. Feature selection is very important in any automated system. To select good features for applying further thresholds, box plots are analyzed. The box plot represents the data in the form of blocks to show its lowest, highest, and median values [35]. The upper adjacent limit is found by:

(17) $$Upperlimit=Q_{3}+\left[1.5\left(Q_{3}-Q_{1}\right)\right]$$

and the lower adjacent limit is found by:

(18) $$Lowerlimit=Q_{1}-\left[1.5\left(Q_{3}-Q_{1}\right)\right]$$

where $Q_{1}$ and $Q_{3}$ are the first and third quartile, respectively. Figure 7 and Figure 8 show the box plot for useful NVD and NVE feature sets, respectively. The box plots are analyzed to find the threshold values. After finding the features and threshold, each candidate region is subjected to that feature set and the threshold is applied. If the feature value fulfills the threshold then it is said to be a true detection; otherwise, it is removed.

### 3.7. Grading of PDR as NVD or NVE

Once the final thresholds are applied and all abnormal blood vessels are detected, the system grades the input image as healthy, NVD, NVE, or both based on the location and distance of these vessels from the OD. If the object detected lies within that one disc distance, defined in Equation (5), it is marked as NVD. If it lies elsewhere, then it is marked as NVE. Table 3 shows the grading criteria of PDR.

## 4. Results

A dataset is a standard tool for the comparisons and evaluation of different algorithms. It is very essential for the proper evaluation of medical image-processing-based algorithms. We evaluated our algorithm on one globally available dataset (MESSIDOR) and one locally available dataset.

MESSIDOR has been established to facilitate computer-aided diabetic retinopathy detection [36]. The images in the dataset were acquired with a TopCon TRC NW6 Non-mydriatic fundus camera with 45° FOV and resolutions of 1440×960, 2240×1488, and 2304×1536 with 8 bits per color plane. A total of 1200 images is contained in this dataset, which is divided into three subsets of 400 images. Each subset is further divided into four parts to facilitate thorough testing. An Excel file accompanies each set that contains medical findings that are used for testing purposes. These images are graded into different categories depending on the number, position, and presence of different lesions. Locally, some data have been collected from the Armed Forces Institute of Ophthalmology (AFIO). A total of 1200 images from the MESSIDOR database and 20 images from the AFIO database is used for the evaluation of the proposed algorithm. A detailed description of the database is given in Table 4.

In order to perform detailed testing, the algorithm is run on the whole database and the results are verified with the help of an ophthalmologist. The results are compared with the ground truth of the abnormal blood vessels marked by ophthalmologists. The results are also verified with the ground truth attached to the database. A detailed evaluation of the proposed system is also performed using different statistical evaluation parameters such as sensitivity, specificity, accuracy, and PPV.

(19) $$Sensitivity=\frac{T_{P}}{\left(T_{P}+F_{N}\right)}$$

(20) $$Specificity=\frac{T_{N}}{\left(T_{N}+F_{P}\right)}$$

(21) $$PPV=\frac{T_{P}}{\left(T_{P}+F_{P}\right)}$$

(22) $$Accuracy=\frac{\left(T_{P}+T_{N}\right)}{\left(T_{P}+T_{N}+F_{P}+F_{N}\right)}$$

where:

- $T_{P}$ are true positives, meaning abnormal blood vessel regions correctly classified as abnormal.
- $T_{N}$ are true negatives, meaning normal blood vessel regions correctly classified an normal.
- $F_{P}$ are false positives, meaning normal blood vessel regions wrongly classified as abnormal.
- $F_{N}$ are false negatives, meaning abnormal blood vessel regions wrongly classified as normal blood vessel regions.

Table 5 shows a comparison of the proposed system with existing methods for PDR detection.

**Figure 9:**
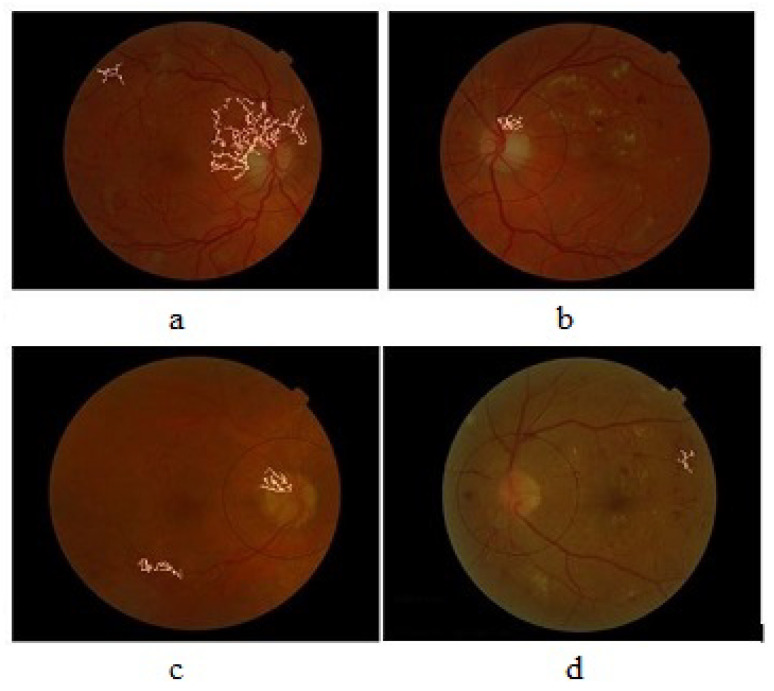
Results of grading of PDR: (**a**) graded as 1 and 2, (**b**) graded as 1, (**c**) graded as 1 and 2, (**d**) graded as 0.

Figure 9 illustrates the abnormal blood vessel detection results for the proposed method. The validity of the proposed system is clearly highlighted. A large number of images is used for the evaluation of the system. The improvement in results is because of the accurate extraction of abnormal blood vessels and the optic disc for the detection of sound feature selection and because of false ROI removal. Table 5 comprehends and compares the results of various state-of-the-art frameworks with our proposed framework. On the basis of accuracy, the proposed algorithm yields the highest accuracy on a large dataset. Other algorithms achieving 0.98 accuracy lack thorough testing on a large dataset. Moreover, unlike the majority of algorithms cited in the related literature, the proposed framework not only highlights the diseased cases, but also categorizes them based on the disease severity, also highlighting the diseased area. Moreover, instead of using deep learning frameworks to obtain accurate results, our algorithm uses basic image-processing techniques to reach the final conclusion, which makes it light and less data-hungry, since it does require pre-training the model, thus giving accurate results even on a small dataset.

## 5. Conclusions

Proliferative diabetic retinopathy (PDR) is an advanced stage of diabetic retinopathy. In this research, a computerized medical system for the screening of PDR is presented. The proposed system performs an analysis of retinal images for grading PDR by analyzing box plots for different sets of features. The proposed system carried out OD detection followed by region-of-interest detection. The abnormal blood vessel detection stage created a binary map of candidate regions using filter banks. A detailed feature set based on the properties of these abnormal blood vessels is created for each candidate region and thresholds are applied to detect all true abnormal blood vessel regions. A further set of features is then applied with an adaptive window and the distribution pattern of true and false detection is analyzed with box plots. By using the coordinates of the OD and the distance of abnormal blood vessels from the optic disc, the system graded the input image into three categories, i.e healthy, NVD, and NVE. The evaluation of the proposed system was performed on the MESSIDOR and AFIO databases. For evaluation, the statistical measures sensitivity, specificity, accuracy, and PPV were used. The results showed that the system achieved an average accuracy of 98.5% and 96.5% for the MESSIDOR and AFIO databases, respectively, while a PPV and specificity of 99.8% was achieved for both databases. This research’s contributions are (i) a complete system for the grading of PDR, (ii) improved results by addressing open issues such as the occurrence of false positives due to the similarity of abnormal blood vessels to normal blood vessels, (iii) the use of two different techniques for the extraction of a useful feature set, based on the properties of normal and abnormal blood vessels, and (iv) the proposal of a method without the need for a classifier, which saves the time required in training.

## Figures and Tables

**Table 1:** Parameter values of Gabor wavelet for NVD.

Parameters	Value for Normal	Value for Abnormal
	Blood Vessels	Blood Vessels
Dilation (a)	11	1.8
Elongation (ϵ)	5	1
Rotation Angle (θ)	10°	10°
$k_{0}$	[0,2.5]	[0,2]

**Table 2:** Parameter values of Gabor wavelet for NVE.

Parameters	Value for Normal	Value for Abnormal
	Blood Vessels	Blood Vessels
Dilation(a)	7	2
Elongation (ϵ)	10	1
Rotation angle (θ)	10°	10°
$k_{0}$	[0,2.5]	[0,2.5]

**Table 3:** Conditions for grading of PDR.

Grade	Condition	Class
0	No abnormal blood vessels present	Healthy
1	A few abnormal blood vessels present 1dd away from the OD	NVE
2	Abnormal blood vessels present within 1dd of OD	NVD

**Table 4:** Database description.

Database	Images	Normal	PDR	NVD	NVE
MESSIDOR	1200	397	37	27	18
AFIO	20	13	7	4	3

**Table 5:** Performance comparison of the proposed system with existing systems for abnormal blood vessel detection.

Sr.	Method	Number of Images	Sen	Spec	Acc/F1 Score
1	Jelinek et al. [13]	27 images	–	–	0.90
2	Saranya et al. [15]	50 images from MESSIDOR and DRIVE	0.96	0.89	0.96
3	Lee et al. [17]	137 images from MESSIDOR	0.96	0.99	0.98
4	Welikala et al. [18]	60 images	0.91	0.92	0.96
5	Garima et al. [15]	799 images	0.95	0.83	0.96
8	Proposed	1200 images from MESIDOR	0.90	1	0.98
	–	20 images from AFIO	0.80	1	0.95

## Data Availability

The MESSIDOR dataset is available at https://www.adcis.net/en/third-party/messidor/ (accessed on 29 June 2023).

## Abbreviations

The following abbreviations are used in this manuscript:

PDR	Proliferative Diabetic Retinopathy
DR	Diabetic Retinopathy
NVD	Neovascularization on Disc
NVE	Neovascularization Elsewhere
SVM	Support Vector Machine
OD	Optic Disc

## References

[1] Leontidis G., Al-Diri B., Hunter A. (2014). Diabetic retinopathy: Current and future methods for early screening from a retinal hemodynamic and geometric approach. Expert Rev. Ophthalmol..

[2] Arden G.B., Ramsey D.J., Kolb H., Fernandez E., Nelson R. (2015). Diabetic retinopathy and a novel treatment based on the biophysics of rod photoreceptors and dark adaptation. Webvision: The Organization of the Retina and Visual System.

[3] Wong T., Cheung C., Larsen M., Sharma S., Simó R. (2016). Diabetic retinopathy. Nat. Rev. Dis. Prim..

[4] Shukla U.V., Tripathy K. (2023). Diabetic Retinopathy. StatPearls.

[5] Nentwich M.M., Ulbig M.W. (2015). Diabetic retinopathy—Ocular complications of diabetes mellitus. World J. Diabetes.

[6] Salamat N., Missen M.M., Rashid A. (2019). Diabetic retinopathy techniques in retinal images: A review. Artif. Intell. Med..

[7] Akram M.U., Khalid S., Tariq A., Javed M.Y. (2013). Detection of Neovascularization in Retinal Images using Multivariate m-Mediods based Classifier. Comput. Med Imaging Graph..

[8] Leontidis G., Al-Diri B., Wigdahl J., Hunter A. Evaluation of geometric features as biomarkers of diabetic retinopathy for characterizing the retinal vascular changes during the progression of diabetes. Proceedings of the 2015 37th Annual International Conference of the IEEE Engineering in Medicine and Biology Society (EMBC).

[9] Mendes L., Marques I.P., Cunha-Vaz J. (2021). Comparison of Different Metrics for the Identification of Vascular Changes in Diabetic Retinopathy Using OCTA. Front Neurosci..

[10] Das S., Kharb a.K., Suchetha M., Raman R., Dhas E. (2021). Deep learning architecture based on segmented fundus image features for classification of diabetic retinopathy. Biomed. Signal Process. Control.

[11] Goatman K.A., Fleming A.D., Philip S., Williams G.J., Olson J.A., Sharp P.F. (2011). Detection of new vessels on the optic disc using retinal photographs. IEEE Trans. Med. Imaging.

[12] Agurto C., Murray V., Barriga E., Murillo S., Pattichis M., Davis H., Russell S., Abramoff M., Soliz P. (2010). Multi-scale AM-FM methods for diabetic retinopathy lesion detection. IEEE Trans Med Imaging.

[13] Jelinek H.F., Cree M.J., Leandro J.J.G., Soares J.V.B., Cesar R.M., Luckie A. (2007). Automated segmentation of retinal blood vessels and identification of proliferative diabetic retinopathy. J. Opt. Soc. Am. A.

[14] Mudigonda S., Oloumi F., Katta K.M., Rangayyan R.M. Fractal analysis of neovascularization due to diabetic retinopathy in retinal fundus images. Proceedings of the E-Health and Bioengineering Conference (EHB), IEEE.

[15] Saranya K.B., Mohideen S.K. A Novel Approach for the Detection of New Vessels in the Retinal Images for screening Diabetic Retinopathy. Proceedings of the 2012 International Conference on Communication and Signal Processing: IEEE Advancing Technology for Humanity.

[16] Roychowdhury S., Koozekanani D.D., Parhi K.K. Automated detection of neovascularization for proliferative diabetic retinopathy screening. Proceedings of the 2016 IEEE 38th Annual International Conference: Engineering in Medicine and Biology Society (EMBC), IEEE.

[17] Lee J., Zee B.C.Y., Li Q. (2013). Detection of Neovascularization Based on Fractal and Texture Analysis with Interaction Effects in Diabetic Retinopathy. PLoS ONE.

[18] Welikalaa R.A., Dehmeshki J., Hoppe A., Tah V., Mann S., Williamson T.H., Barman S.A. (2014). Automated detection of proliferative diabetic retinopathy using a modified line operator and dual classification. Comput. Methods Programs Biomed..

[19] Yu S., Xiao D., Kanagasingam Y. Automatic detection of neovascularization on optic disk region with feature extraction and support vector machine. Proceedings of the 2016 IEEE 38th Annual International Conference of the Engineering in Medicine and Biology Society (EMBC), IEEE.

[20] Coelho D.F.G., Rangaraj M.R., Vassil S.D. Detection of neovascularization near the optic disk due to diabetic retinopathy. Proceedings of the 2016 24th European Signal Processing Conference (EUSIPCO), IEEE.

[21] Yu S., Xiao D., Kanagasingam Y. (2018). Machine learning based automatic neovascularization detection on optic disc region. IEEE J. Biomed. Health Inform..

[22] Argyrios C., Hurtut T., Cheriet F. Proliferative diabetic retinopathy characterization based on the spatial organization of vascular junctions in fundus images. Proceedings of the 2017 IEEE 14th International Symposium on Biomedical Imaging (ISBI 2017), IEEE.

[23] Leeza M., Farooq H. (2019). Detection of Severity Level of Diabetic Retinopathy Using Bag of Features Model. IET Comput. Vis..

[24] Zhang L., Li Q., You J., Zhang D. (2009). A modified matched filter with double-sided thresholding for screening proliferative diabetic retinopathy. IEEE Trans. Inf. Technol. Biomed..

[25] Qummar S., Khan F.G., Shah S., Khan A., Shamshirb S., Rehman Z.U., Khan I.A., Jadoon W. (2019). A Deep Learning Ensemble Approach for Diabetic Retinopathy Detection. IEEE Access.

[26] Elgafi M., Sharafeldeen A., Elnakib A., Elgarayhi A., Alghamdi N.S., Sallah M., El-Baz A. (2022). Detection of Diabetic Retinopathy Using Extracted 3D Features from OCT Images. Sensors.

[27] Mehboob A., Akram M.U., Alghamdi N.S., Abdul Salam A. (2022). A Deep Learning Based Approach for Grading of Diabetic Retinopathy Using Large Fundus Image Dataset. Diagnostics.

[28] Tang M.C., Teoh S.S., Ibrahim H., Embong Z. (2021). Neovascularization detection and localization in fundus images using deep learning. Sensors.

[29] Tang M.C., Teoh S.S., Ibrahim H., Embong Z. (2022). A deep learning approach for the detection of neovascularization in fundus images using transfer learning. IEEE Access.

[30] Lavanya S., Naveen P. (2022). Detection of Retinal Neovascularization Using Optimized Deep Convolutional Neural Networks. J. Trends Comput. Sci. Smart Technol..

[31] Tariq A., Akram M.U. An Automated System for Colored Retinal Image Background and Noise Segmentation. Proceedings of the 2010 IEEE Symposium on Industrial Electronics and Applications (ISIEA).

[32] Antoine J.P., Carette P., Murenzi R., Piette B. (1993). Image analysis with two-dimensional continuous wavelet transform. Signal Process..

[33] Akram M.U., Khan S.A. (2011). Multilayered thresholding-based blood vessel segmentation for screening of diabetic retinopathy. Eng. Comput..

[34] Usman A., Abbas S., Akram M.U., Nadeem Y. (2014). A Robust Algorithm for Optic Disc Segmentation from Colored Fundus Images: Image analysis and Recognition.

[35] Nelson L.S. (1989). Evaluating Overlapping Confidence Intervals. J. Qual. Technol..

[36] MESSIDOR. https://www.adcis.net/en/third-party/messidor/.

